# Increased platelet-lymphocyte ratio closely relates to inferior clinical features and worse long-term survival in both resected and metastatic colorectal cancer: an updated systematic review and meta-analysis of 24 studies

**DOI:** 10.18632/oncotarget.16020

**Published:** 2017-03-08

**Authors:** Nan Chen, Wanling Li, Kexin Huang, Wenhao Yang, Lin Huang, Tianxin Cong, Qingfang Li, Meng Qiu

**Affiliations:** ^1^ West China School of Medicine/West China Hospital, Sichuan University, Chengdu, China; ^2^ Department of Medical Oncology, Cancer Center, the State Key Laboratory of Biotherapy, West China Hospital, Sichuan University, Chengdu, Sichuan, China

**Keywords:** colorectal cancer, platelet-lymphocyte ratio, prognosis, clinical features, meta-analysis

## Abstract

Colorectal cancer (CRC) is one of the most common cancers worldwide. However, the prognostic and clinical value of platelet-lymphocyte ratio (PLR) in colorectal cancer was still unclear, which attracted more and more researchers considerable attention. We performed a systematic review and meta-analysis to investigate the relationship between PLR and survival as well as clinical features of CRC update to September 2016. The hazard ratio (HR) or odds ratio (OR) with 95% confidence interval (CI) were calculated to access the association. We included 24 eligible studies with a total of 13719 patients. Elevated PLR predicted shorter overall survival (OS) (HR=1.47; 95%CI, 1.28-1.68; *p*<0.001), poorer disease-free survival (DFS) (HR=1.51; 95% CI, 1.2-1.91; *p*=0.001), and worse recurrence-free survival (RFS) (HR=1.39; 95% CI, 1.03-1.86; *p*=0.03), but had nothing to do with Cancer-specific survival (CSS) (HR=1.14; 95% CI, 0.92-1.42; *p*=0.223). After trim and fill method, the connection between PLR and DFS disappeared (HR=1.143; 95%CI, 0.903-1.447; *p*=0.267). By subgroup analyze, we found that increased PLR predicated a worse OS and DFS in patients who underwent surgery, and this prognostic role also shown both in metastatic and nonmetastatic patients. In addition, elevated PLR was associated with poorly differentiated tumor (OR=1.51; 95% CI, 1.26-1.81; *p*<0.001), higher tumor stage (OR=1.25; 95% CI, 1.05-1.49; *p*=0.012), lymphovascular invasion (LVI) (OR=1.25; 95% CI, 1.09-1.43; *p*=0.001), and the recurrence of CRC (OR=2.78; 95% CI, 1.36-5.68; *p*=0.005). We indicated that pretreatment PLR was a good prognostic marker for CRC patients. High PLR was related to worse OS, RFS and poor clinical characteristics.

## INTRODUCTION

Colorectal cancer (CRC) caused almost 700,000 deaths worldwidely every year, making it the world's fourth most deadly cancer [1]. It was the third most commonly diagnosed cancer in males and the second in females, with an estimated 1.4 million cases and 693,900 deaths occurring in 2012 [2]. The lifestyle changes in past low-CRC-risk countries resulted in rapid growth of colorectal cancer and the 5-year survival rate was still poor despite the progress of the treatment [3–6]. Pretreatment predicting indexes are in dire need to forecast potential of the tumor recurrence and prognosis for that the majority of the available prognostic markers are assessed postoperatively. The clinical and pathological TNM stages, the number of resected lymph nodes (nLNs), carcino-embryonic antigen (CEA), the lymphovascular invasion (LVI), the perineural invasion, in addition to some molecular markers (eg. PinX1, RAS, BRAF, MMR and so on) have all been identified as prognostic factors [7–11], however several weaknesses limited their application in routine clinical practice, such as high costs, lack of standardization, low consistency, and poor reproducibility [7, 12, 13, 14]. Therefore, finding a proper prognostic factors to assist coloractal cancer patients in guiding appropriate treatment to improve the therapeutic effectiveness is extremely urgent.

Prior studies showed that systemic inflammatory response (SIR) status played a vital role in tumor progression and therapeutic response [15–19]. The levels of platelets and lymphocytes represents the systemic inflammatory response (SIR), and they are easily obtained by widely used peripheral blood test. Platelet-lymphocyte ratio (PLR) as a combination of these two factors has been reported to be associated with poor prognosis in different tumor types, including CRC [20–32], but some other studies drew a different conclusion [33–43]. In brief, independent research results of the relationship between PLR and its impact on survival and clinical features were still inconsistent, partly due to limited published studies previously, various confounding factors and less detailed analysis. Recently, a lot of new studies on this issue were published continuously. Thus the aim of our study was to perform a systemic review and meta-analysis with all eligible current evidence to clarify this relationship and to evaluate whether PLR was an independent risk factors for the prognosis of patients with colorectal cancer.

## RESULTS

### Literature search and study selection

**Figure 1:**
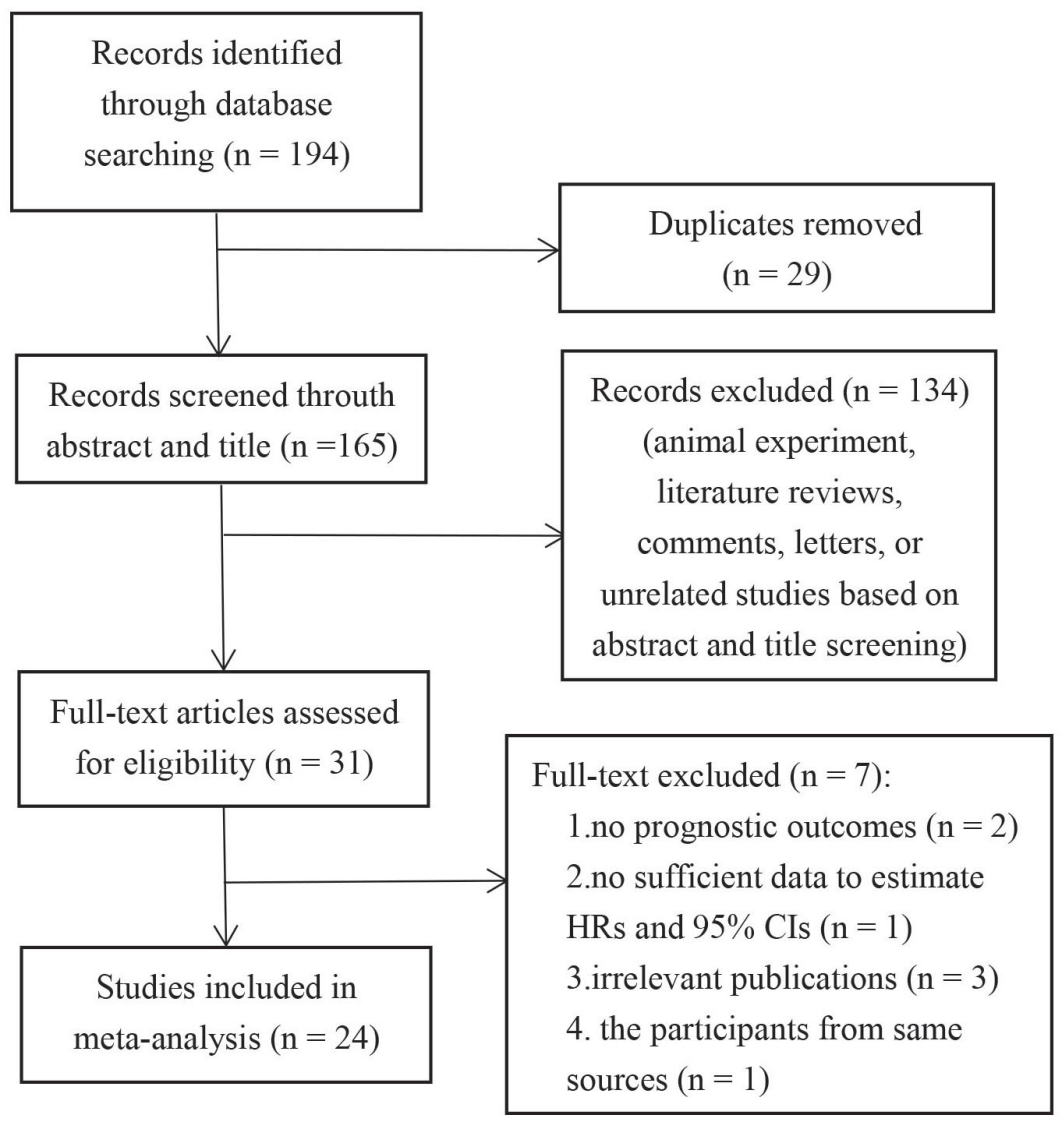
Flow chat of literature search and study selection

A total of 194 relevant publications were initially retrieved. Of these, 29 duplicates were removed, 134 publications were excluded because the studies were animal experiment, literature reviews, comments, letters, or unrelated studies based on the titles and abstracts screening. After reading the full text, 7 publications were excluded due to irrelevant publications, studies with overlapping case series or lack of sufficient data for analysis, Therefore, a total of 24 publications with 13719 patients were included [20–43]. All of these studies contained the required information and evaluated the correlation between PLR and the prognosis of CRC. Figure 1 presents a summary of the study selection process.

### Study characteristics

The main features of the 24 selected studies are shown in Table 1. From the 24 studies, fourteen publications were originated from the Asian (eight from China, three from Korea, three from Japan), nine were performed in Caucasian population (four from the UK, one from Hungary, one from Canada, one from Australia, one from Austria, one from Italy), and one from USA with mixed races. The OS was investigated in 22 studies, the DFS rate was analyzed in 12 studies, the CSS rate was evaluated in 4 studies, and the RFS rate was reported in 2 studies. These eligible studies were published from 2012 to 2016. Thirteen of these directly provided HR in multivariate analysis, and survival data of nine studies were extracted from univariate analysis while survival data of two studies were extracted from survival curves. The extracted data in detail were presented in Supplementary Table S1 and S2, while detailed NOS scores of each included study were presented in Supplementary Table S4.

**Table 1:** Study characteristics

Author	Year	Country	Ethnicity	Location	Na	Sex(male/female)	Stage	Metastasis	Treatmentb	Survival analysis	Analysis	NOS score
Azab	2014	USA	Mixed	Colorectal cancer	580	273/307	I/II/III/IV	Y	Surgery	OS/DFS	M/M	8
Baranyai	2014	Hungary	Caucasian	Colorectal cancer	336	180/156	I/II/III/IV	N	Surgery	OS/DFS	U/U	5
Baranyai	2014	Hungary	Caucasian	Colorectal cancer	118	80/38	IV	Y	Surgery	OS	U	5
Carruthers	2012	UK	Caucasian	Rectal cancer	115	75/40	I/II/III	Y	Surgery	OS/DFS	U/U	6
Chan	2016	Australia	Caucasian	Colorectal cancer	1623	801/882	I/II/III	N	Surgery	OS	U	8
Choi	2015	Canada	Caucasian	Colorectal cancer	549	296/253	I/II/III	N	Surgery	OS/RFS	U/U	8
He	2013	China	Asian	Colorectal cancer	243	155/88	IV	Y	Non surgery	OS	M	8
Kwon	2012	Korea	Asian	Colorectal cancer	200	123/77	I/II/III/IV	Y	Surgery	OS	M	8
Li	2016	China	Asian	Rectal cancer	140	81/59	I/II/III	N	Surgery	OS/DFS	U/U	7
Li	2016	China	Asian	Colorectal cancer	5336	3167/2169	I/II/III	Y	Surgery	OS/DFS	M/M	6
Li	2015	China	Asian	Colon cancer	110	58/52	IV	Y	Surgery	OS	M	7
Mori	2015	Japan	Asian	Colorectal cancer	157	87/65	I/II/III	N	Surgery	DFS	U	6
Neal	2015	UK	Caucasian	Colorectal cancer	302	192/110	IV	Y	Surgery	OS/CSS	U/U	7
Neofytou	2014	UK	Caucasian	Colorectal cancer	140	88/52	IV	Y	Surgery	OS/DFS	M/M	9
Neofytou	2015	UK	Caucasian	Colorectal cancer	140	88/52	IV	Y	Surgery	CSS	U	9
Ozawa	2015	Japan	Asian	Colorectal cancer	234	142/92	II	N	Surgery	DFS/CSS	M/M	7
Passardi	2016	Italy	Caucasian	Colorectal cancer	289	174/115	I/II/III/IV	Y	Non surgery	OS	M/	7
Son	2013	Korea	Asian	Colon cancer	624	368/256	I/II/III	N	Surgery	OS/DFS	M/M	7
Song	2015	Korea	Asian	Colorectal cancer	177	83/94	IV	N	Non surgery	OS	U	5
Sun	2014	China	Asian	Colon cancer	255	135/120	I/II/III	N	Surgery	OS/DFS	M/M	7
Szkandera	2014	Austria	Caucasian	Colon cancer	372	217/155	II/III	N	Surgery	OS	M	7
Toiyama	2013	Japan	Asian	Rectal cancer	84	62/22	I/II/III	N	Surgery	OS/DFS	U/U	6
Ying	2014	China	Asian	Colorectal cancer	205	144/61	I/II/III	N	Surgery	OS/CSS/RFS	M/M/M	7
You	2016	China	Asian	Colorectal cancer	1314	785/529	I/II/III/IV	Y	Surgery	OS	M	6
Zou	2016	China	Asian	Colorectal cancer	216	137/79	I/II/III/IV	Y	Surgery	OS/DFS	M/M	7

^a^ Number of included patients.

^b^Unsurgery includes patients undergoing chemotherapy, chemoradiotherapy, or other treatment, but not doing surgery. Surgery including patients getting surgery with or without other treatment.

Abbreviations: NA: not available; OS: overall survival; DFS: disease-free survival; CRM: cancer-ralated mortality; TLR: Time to local recurrence; RFS: recurrence-free survival; CSS: Cancer-specific survival; M: multivariate analysis; U: univariate analysis; NOS: Newcastle-Ottawa Quality Assessment Scale; PLR: platelet-lymphocyte ratio.

### Prognostic value of PLR for CRC patients

**Figure 2:**
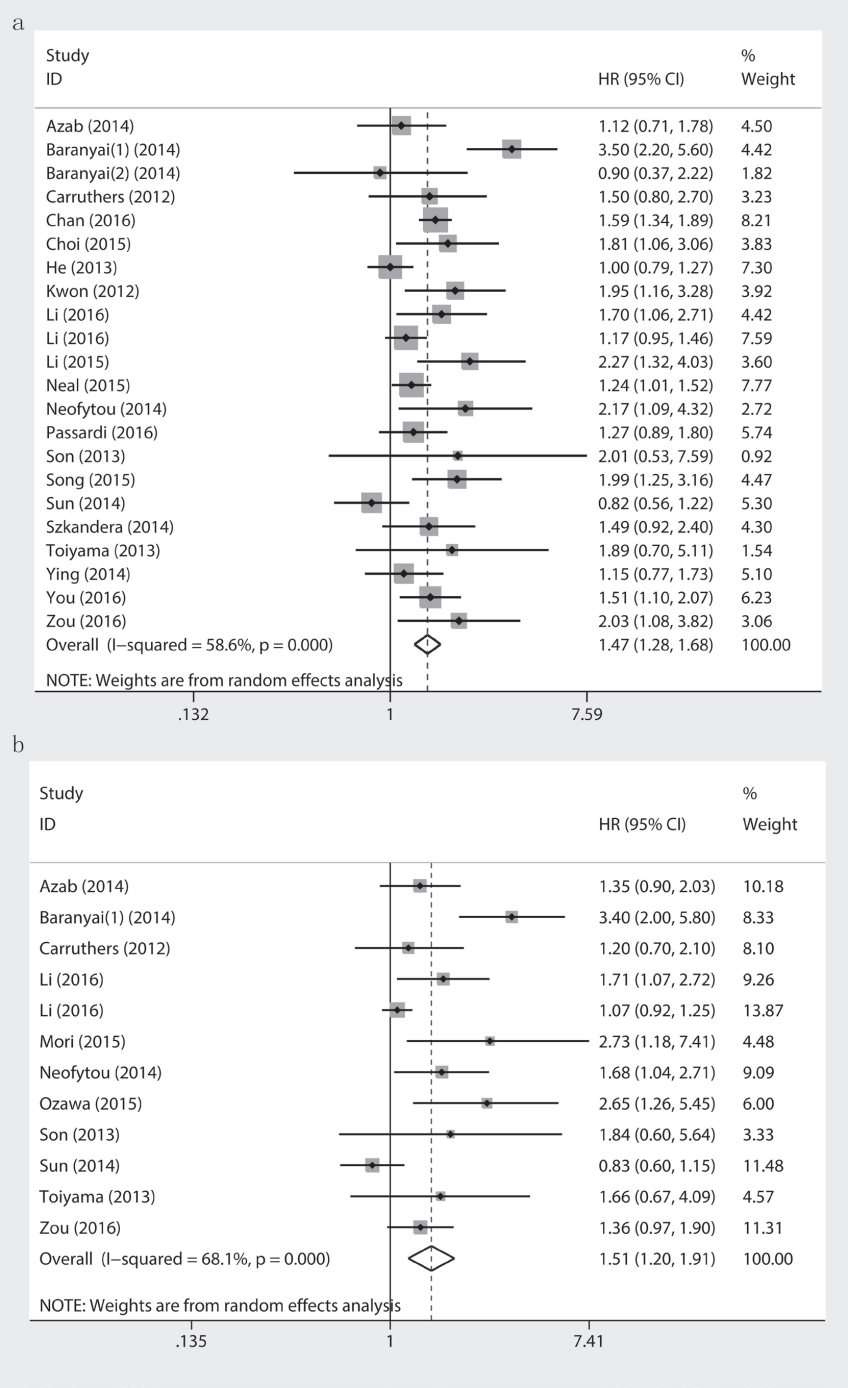
Results of prognostic analysis for PLR in CRC for OS **a**. and DFS **b**.

Twenty-two studies containing 13328 CRC patients reported the impact of PLR on OS, which showed the existence of heterogeneity across the studies (I^2^ = 58.6%, Ph < 0.001). We detected that higher PLR predicate shorter OS for CRC patients (HR = 1.47; 95%CI, 1.28-1.68; *p* < 0.001)(Table 2, Figure 2a). Furthermore, twelve studies containing 8217 CRC patients suggested that elevated PLR was significantly associated with a poor DFS (HR = 1.51; 95% CI, 1.20-1.91; *p* = 0.001) (Figure 2b). Increased PLR predicated a worse RFS (HR = 1.39; 95% CI, 1.03-1.86; *p* = 0.001) in the combination of 2 studies containing 754 CRC patients, however it was not related to CSS (HR = 1.14; 95% CI, 0.92-1.42; *p* = 0.223) (Table 2) in the combination of four studies containing 881 CRC patients.

**Table 2:** The pooled data on survival of meta-analysis

Variables	*N*^a^	Case^b^	Pooled data		Heterogeneity		*N*^a^	Case^b^	Pooled data		Heterogeneity	
			HR(95%CI)	*P*	*I*^2^	*Ph*			HR(95%CI)	*P*	*I*^2^	*Ph*
	Overall survival						Disease-free survival					
Overall	22	13328	1.47(1.28,1.68)	<0.001	58.60%	<0.001	12	8217	1.51(1.2,1.91)	0.001	68.10%	<0.001
**By ethnicity**												
Caucasian	9	3844	1.6(1.3,1.96)	<0.001	60.00%	0.01	3	591	1.9(1.06,3.4)	0.031	73.40%	0.023
Asian	12	8904	1.41(1.17,1.7)	0.001	57.00%	0.008	8	7046	1.37(1.06,1.78)	0.017	60.80%	0.013
Mixed	1	580	1.12(0.71,1.77)	0.629	/	/	1	580	1.35(0.9,2.03)	0.148	/	/
**By analysis method**												
Univariate	10	3687	1.57(1.26,1.96)	<0.001	70.70%	<0.001	5	832	1.96(1.31,2.94)	0.011	50.90%	0.087
Multivariate	12	9641	1.32(1.17,1.48)	<0.001	41.00%	0.068	7	7385	1.27(1.02,1.6)	0.036	57.50%	0.028
**By treatment**												
Surgery	19	12619	1.51(1.3,1.74)	<0.001	55.1%	0.002	12	8217	1.51(1.2,1.91)	0.001	68.10%	<0.001
Non surgery	3	709	1.31(0.9,1.89)	0.157	71.2%	0.031						
**By cut-off**												
Single cut-off	15	10257	1.61(1.36,1.89)	<0.001	47.90%	0.02	10	7382	1.69(1.28,2.22)	0.001	67.70%	0.001
Multiple cut-offs	7	3071	1.27(1.03,1.56)	0.026	62.50%	0.014	2	835	1.04(0.65,1.67)	0.856	69.50%	0.07
<200	7	1043	1.50(1.24,1.81)	<0.001	3.40%	0.4	6	870	1.72(1.34,2.2)	<0.001	<0.01%	0.553
≥200	15	12245	1.44(1.22,1.71)	<0.001	68.10%	<0.001	6	7347	1.36(0.99,1.87)	0.059	77.90%	<0.001
**By sample size**												
<200	7	884	1.8(1.44,2.26)	<0.001	<0.01%	0.698	5	636	1.62(1.2516,2.11)	<0.001	<0.01%	0.653
≥200	15	12444	1.39(1.19,1.63)	<0.001	66.00%	<0.001	7	7581	1.46(1.06,2.01)	0.019	78.10%	<0.001
**By study result**												
Positive	11	5007	1.57(1.41,1.74)	<0.001	12.80%	0.325	5	887	1.65(1.32,2.05)	<0.001	2.50%	0.392
Negative	11	8221	1.29(1.05,1.59)	0.016	62.50%	0.002	7	7330	1.36(0.99,1.87)	0.058	73.20%	0.001
**By metastatic**												
Yes	12	8963	1.34(1.16,1.54)	<0.001	38.70%	0.083	5	6387	1.17(1.04,1.33)	0.012	13.80%	0.327
No	10	4365	1.62(1.27,2.06)	0.001	65.30%	0.002	7	1830	1.89(1.15,3.08)	0.011	77.00%	<0.001
**By location**												
Colorectal cancer	15	11628	1.47(1.26,1.71)	<0.001	64.40%	<0.001	7	6999	1.71(1.23,2.37)	0.001	76.80%	<0.001
Rectal cancer	3	339	1.65(1.17,2.34)	0.005	<0.01%	0.914	3	339	1.5(1.07,2.08)	0.017	<0.01%	0.615
Colon cancer	4	1361	1.49(0.92,2.4)	0.183	69.40%	0.02	2	879	0.89(0.65,1.21)	0.45	42.70%	0.186
**By NOS**												
<6	3	631	2.02(1.06,3.87)	0.034	74.00%	0.021	1	336	3.4(2.0,5.79)	<0.001	/	/
≥6	19	12597	1.34(1.24,1.45)	<0.001	43.50%	0.023	11	7881	1.35(1.11,1.65)	0.002	50.90%	0.026
	**Cancer-specific survival**						**Recurrence-free survival**					
Overall	4	881	1.14(0.92,1.42)	0.223	63.70%	0.041	2	754	1.39(1.03,1.86)	0.03	13.50%	0.282

^a^ Numbers of studies included in the meta-analysis.

^b^ Number of patients of included studies.

Abbreviations: NA: not available; HR: hazard ratio; 95%CI: confidence interval; *P*: *p* value of pooled HR; *I*^2^: value of Higgins I-squared statistics; *Ph*: *p* value of Heterogeneity test.

**Figure 3:**
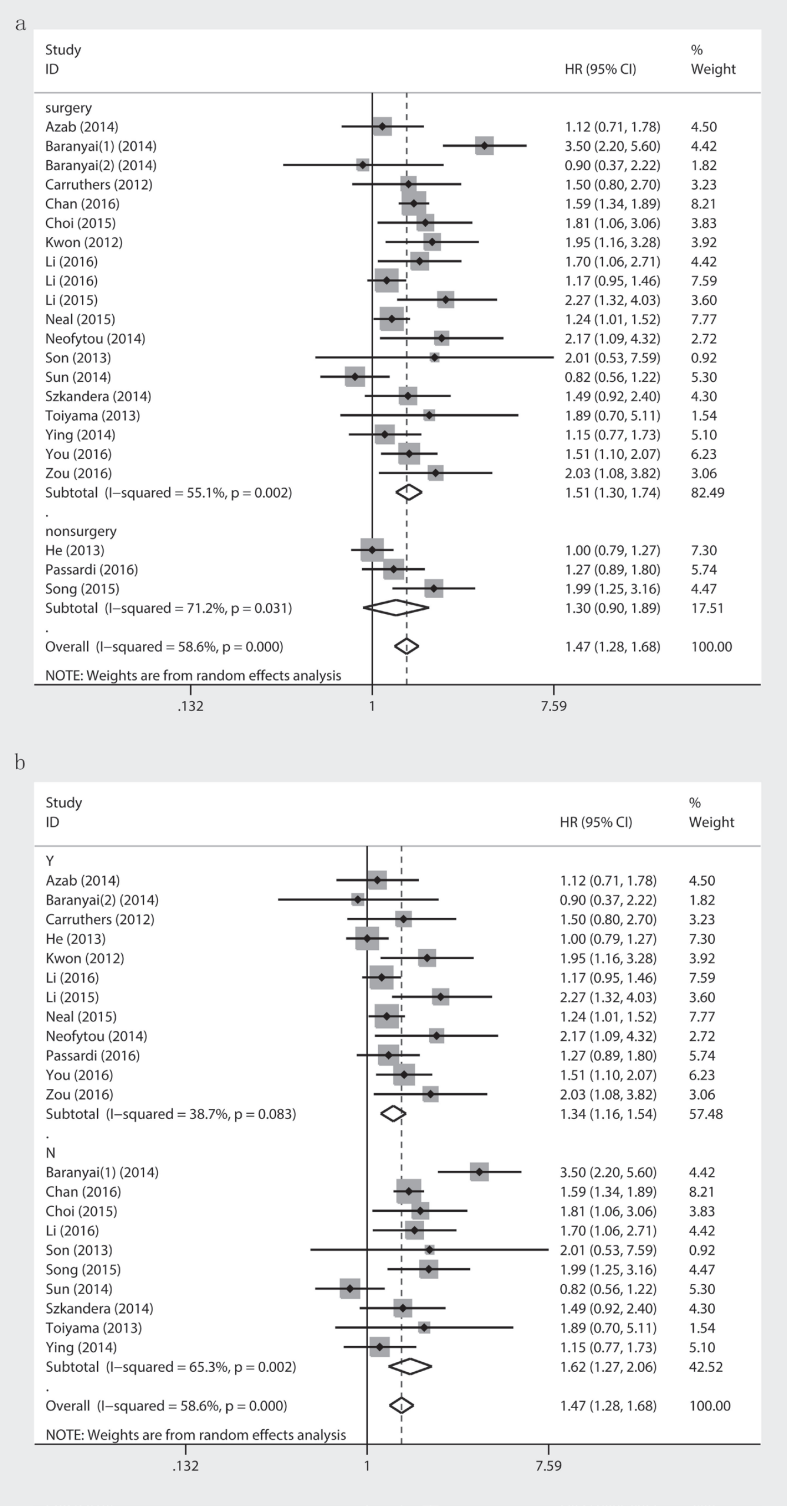
Association between PLR and OS stratified by treatment **a**., metastatic **b**.

To explain the source of heterogeneity, we further performed a subgroup analysis by ethnicity, analysis method, major treatment therapy, respective cut-off value, sample size, metastasis status, tumor location and NOS score. The higher PLR was, the shorter OS and DFS were showed both in Caucasian ( [OS: HR = 1.6; 95% CI, 1.3-1.96; *p* < 0.001]; [DFS: HR = 1.9; 95% CI, 1.06-3.4; *p* = 0.031]) and Asian groups ( [OS: HR = 1.41; 95% CI, 1.17-1.7; *p* = 0.001]; [DFS: HR = 1.37; 95% CI, 1.06-1.78; *p* = 0.017]). Significant association were detected whether univariate analysis ( [OS: HR = 1.57; 95% CI, 1.26-1.96; *p* < 0.001]; [DFS: HR = 1.96; 95% CI, 1.31-2.94; *p* = 0.011]) or multivariate ( [OS: HR = 1.32; 95% CI, 1.17-1.48; *p* < 0.001]; [DFS: HR = 1.27; 95% CI, 1.02-1.6; *p* = 0.036]) analysis were used in original studies. Elevated PLR was strongly associated with poor OS in patients who underwent surgical resection ( [OS: HR = 1.51; 95% CI, 1.3-1.74; *p* < 0.001]), but not in nonsurgery subgroup which involved limited studies ( [OS: HR = 1.31; 95% CI, 0.9-1.89; *p* = 0.157]) (Figure 3a). After enlarging the sample size by meta-analysis, we overthrow the old conclusion in the negative study result subgroup that PLR had nothing to do with OS. For metastatic colorectal cancer patients, increased PLR predicated a worse OS and DFS ( [OS: HR = 1.34; 95% CI, 1.16-1.54; *p* < 0.001]; [DFS: HR = 1.17; 95% CI, 1.04-1.33; *p* = 0.012]), and this prognostic implication also existed in nonmetastatic CRC ( [OS: HR = 1.62; 95% CI, 1.27-2.06; *p* = 0.001]; [DFS: HR = 1.89; 95% CI, 1.15-3.08; *p* = 0.011]) (Figure 3b). Significant association were almost detected in all stratified analysis which further proved our results. More details about the subgroup analysis of OS and DFS were presented in Table 2.

### PLR and clinical characteristics of CRC patients

**Figure 4:**
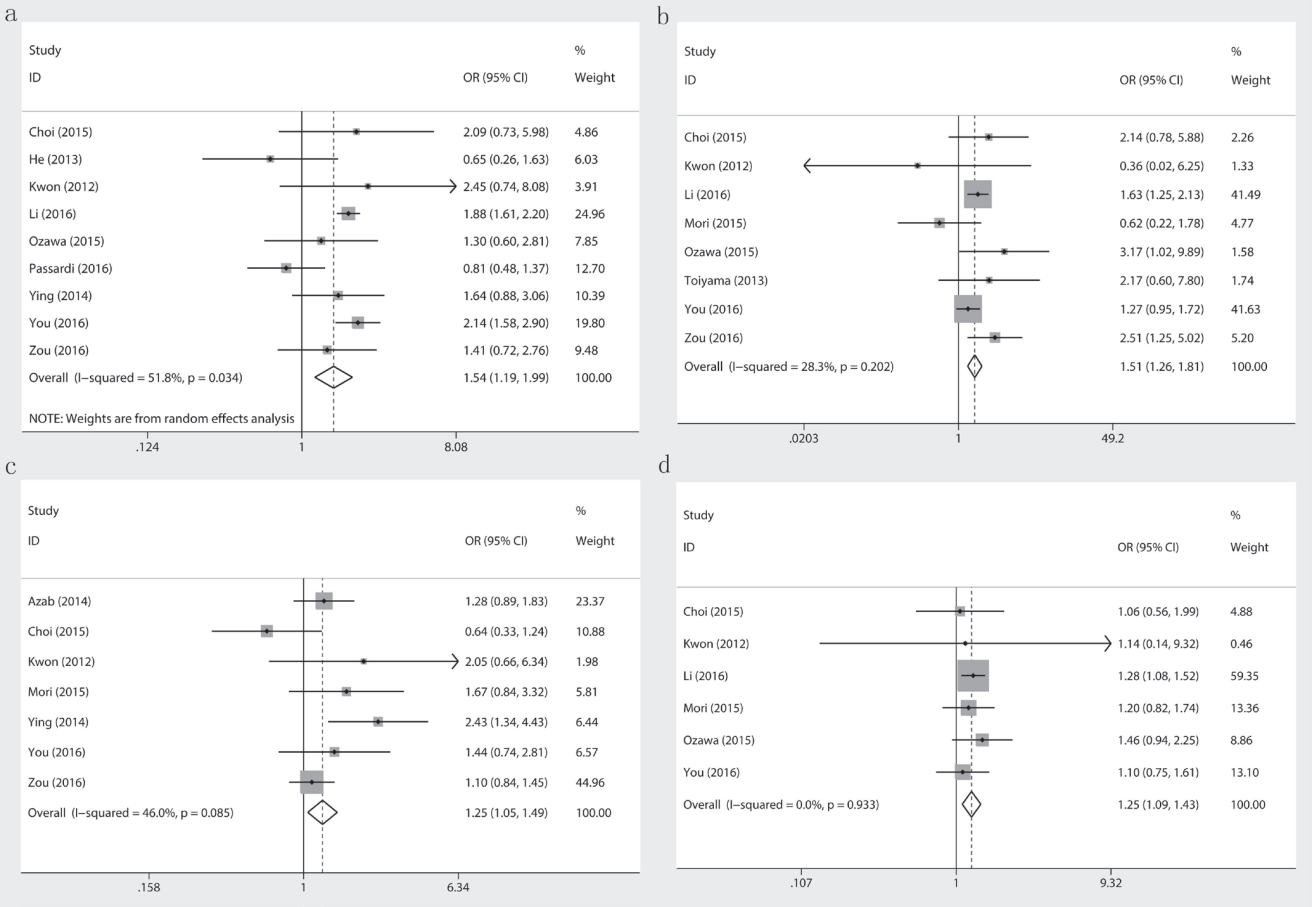
Association between PLR and tumor location **a**., differentiation **b**., stage **c**. and lymphovascular invasion **d**.

In addition, we examined the association between PLR and the clinical parameters of colorectal cancer (Table 3). Peripheral higher PLR was detected to be associated with gender(OR = 0.8; 95% CI, 0.72-0.90; *p* < 0.001), cancer location(OR = 1.54; 95% CI, 1.19-1.99; *p* = 0.001), poorer differentiation status (OR = 1.51; 95% CI, 1.26-1.81; *p* < 0.001), higher tumor stage(OR = 1.25; 95% CI, 1.05-1.49; *p* = 0.012), higher T (OR = 2.13; 95% CI, 1.36-3.34; *p* = 0.001) stage and N stage(OR = 1.35; 95% CI, 1.17-1.54; *p* < 0.001), more lymphovascular invasion (OR = 1.25; 95% CI, 1.09-1.43; *p* = 0.001), and recurrence(OR = 2.78; 95% CI, 1.36-5.68; *p* = 0.005) in colorectal cancer patients (Figure 4).

**Table 3:** The pooled data on clinical characteristics of included studies

Variables	*N*^a^	Case^b^	Pooled data		Heterogeneity	
			OR(95%CI)	*P*	*I*^2^	*Ph*
**Gender**	13	9483				
Female		3908	Reference			
Male		5575	0.8(0.72,0.90)	<0.001	<0.01%	0.512
**Location**	9	8262				
Rectaum		4261	Reference			
Colon		4001	1.54(1.19,1.99)	0.001	51.80%	0.034
**Differentiation**	8	7388				
Well and moderately		6526	Reference			
Poorly		862	1.51(1.26,1.81)	<0.001	28.30%	0.202
**Stage**	7	3156				
I/II		1770	Reference			
III/IV		1386	1.25(1.05,1.49)	0.012	46.00%	0.085
**T**	7	6419				
1,2		1516	Reference			
3,4		4903	2.13(1.36,3.34)	0.001	51.10%	0.056
**N**	6	6583				
Negative(N0)		3504	Reference			
Positive(N1,2)		3079	1.35(1.17,1.54)	<0.001	22.80%	0.262
**LVI(lymphovascular invasion)**	6	7951				
No		5733	Reference			
Yes		2218	1.25(1.09,1.43)	0.001	<0.01%	0.933
**Recurrence**	2	236				
Absent		192	Reference			
Present		44	2.78(1.36,5.68)	0.005	<0.01%	0.352
**Chemotherapy**	4	6670				
No		2214	Reference			
Yes		4456	1.09(0.74,1.61)	0.674	72.00%	0.013

^a^ Numbers of studies included in the meta-analysis.

^b^ Number of patients of included studies.

Abbreviations: LVI: lymphovascular invasion; OR: odds ratio; 95%CI: confidence interval; *P*: *p* value of pooled HR; *I*2: value of *X*2 based I-squared statistics; *Ph*: *p* value of Heterogeneity test.

### Sensitivity analysis and publication bias

**Figure 5:**
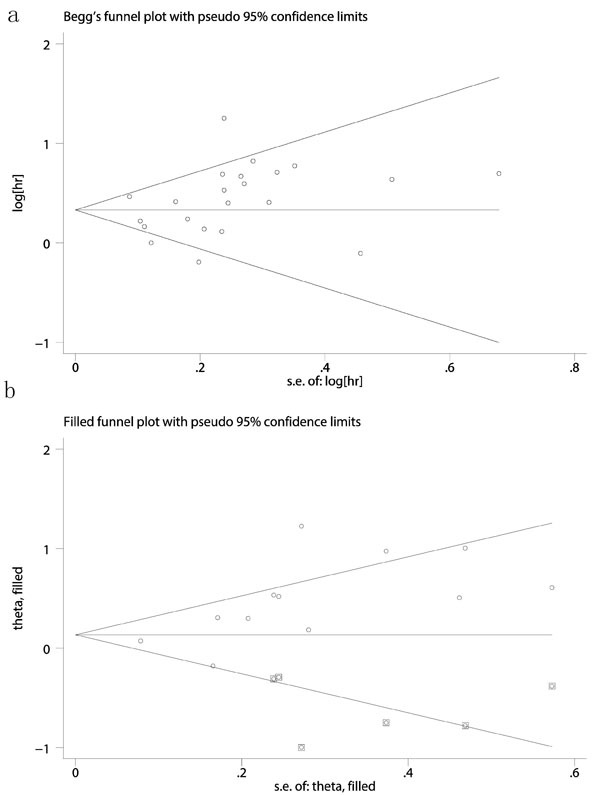
Egger's test for accessing publication biases for role of PLR on OS **a**. and DFS (**b**., after trim and fill method).

To identify the source of heterogeneity across selected studies, sensitivity analysis was conducted by removing each study in turn from the analysis. The pooled ORs and HRs were not significantly changed, indicating the stability of our analyses. The funnel plots were largely symmetrical for OS in patients with CRC, and the results of the Begg's and Egger's tests showed no evidence of significant publication bias among the included studies (OS: Begg's test Pr > |z| = 0.159, Egger's test *P* > |t| = 0.130) (Figure 5a). But slight publication bias was seen in DFS (DFS: Begg's test Pr > |z| = 0.064, Egger's test *P* > |t| = 0.013). So a trim and fill method was used to estimate the asymmetry in the funnel plot (HR = 1.143; 95%CI, 0.903-1.447, *p* = 0.267)(Figure 5b).

## DISCUSSION

This systematic review and meta-analysis, including 24 individual studies of 13719 patients, found that increased PLR was strongly associated with poor overall survival and recurrence-free survival in patient with colorectal cancer. However, PLR was unrelated to cancer-specific survival and disease-free survival after trim and fill method. The stratified analyses showed that elevated PLR was associated with poor outcome in both Caucasian and Asian population, univariate and multivariate analysis, metastatic and nonmetastatic CRC, and resected patients. However, we did not observe the significant association in nonsurgery subgroup for that the number of the included original studies in this subgroup is limited. For the negative study result subgroup, the HR of OS was 1.29 (95% CI, 1.05-1.59; *p* = 0.016), which meaned that after enlarging the sample size by meta-analysis, we overthrow the old conclusion that PLR had nothing to do with OS. Our finding confirmed the hypothesis that PLR was an appropriate prognostic factor for CRC patient survival.

Cancer progression and prognosis was determined not only by tumor characteristics but also by the host inflammatory response [44, 45]. Using clinical, inflammatory, and molecular biomarkers as CRC prognostic factors are increasingly interesting, but there remained a lack of reliable, reproducible, and low-cost markers that can be readily incorporated into routine practice to optimally predict prognosis and guide treatment [31]. Some combinations of the inflammatory response parameters (eg. lymphocytes, neutrophils, platelets and acute-phase proteins, which are simple and easy to measure using standardized and widely used assays) including platelet-to-lymphocyte ratio (PLR), neutrophil-to-lymphocyte ratio (NLR), lymphocyte-to-monocyte ratio (LMR) and albumin/globulin ratio (AGR), have been performed to evaluate the prognosis in various cancers, including CRC, and so on [27, 33, 46–48]. There were many reasons for PLR's ideal prognostic role in CRC patient. Firstly, platelets secrete several tumor growth and angiogenic factors, which might influence tumor progression [49]. Secondly, while in antitumor reaction of the immune system, the CD8+ and CD4+ T-lymphocyte interaction among each other can induce tumor cell apoptosis, which can improve the survival of CRC patients for the chemotherapy efficacy [50]. These supported our finding that the PLR was a promising prognostic factors for the survival of CRC patients, which was consistent with previous meta-analysis [51–54], however, our study was to some extent superior to the previous studies because of much more included studied and patients, more detailed analyses and less limitation. We included all current eligible relative studies by systemic review and meta-analysis. We did subgroup analyses to explore the heterogeneity sources, besides we explored the relationship between PLR and the inferior clinical features.

By analyzing clinical factors, we found the relationship between the increased PLR and the clinical characteristics of CRC patients. PLR tended to be higher in colon cancer than rectum which need further explanation. Poorly differentiated cancer always accompanied with elevated PLR, for that poorly differentiated tumour cells growing faster with angiogenic and tumour growth factors secreted by platelet cell, such as platelet factor 4 (PF4), thrombospondin, vascular endothelial growth factor (VEGF), transforming growth factor beta (TGF-β) and platelet-derived growth factor (PDGF) [36]. Moreover, platelets reflected the invasive potential of CRC and was closely associated with lymphovascular invasion (LVI) [55]. PLR was a good prognostic marker in mCRC patients, because several studies have shown that platelets induce circulating tumor cell epithelial-mesenchymal transition and promote extravasation to metastatic sites [43, 56, 57]. Lymphocytes were involved in cancer immune surveillance which influenced the tumor recurrence to some extent [58]. Our study results indicated that the relationship between PLR and some clinical factors presented a new researching direction for future research. Moreover, the easily got PLR can be used to reflect some clinical characteristics which were difficult to obtain like tumour differentiation, lymphovascular invasion (LVI), recurrence and so on. Pretreatment blood test for PLR played a vital role in assessment of cancer characteristics and patients prognosis.

There were limitations in our systematic review. First, the included studies were almost retrospective studies and more studies with prospective design were warranted in future. Second, eleven enrolled studies applied univariate analysis only (without providing multivariate analysis data), while subgroup analysis showed the prognostic values of PLR existed in these studies. Moreover, there are significant heterogeneity existing in OS and DFS analysis. Therefore additional large cohorts of prospective studies are needed to correct for heterogeneity.

In conclusion, peripheral blood PLR was an effective prognostic marker for CRC patients. Elevated PLR was related to worse overall survival and recurrence-free survival, but not for disease-free survival and cancer-specific survival. The prognostic utility of PLR might help to guide use of individual therapies and patient counselling in future.

## MATERIALS AND METHODS

### Search strategy

PubMed, Web of Science and Embase were searched from inception to September 2016. The search strategy used the keywords as follows: “PLR” or “platelet lymphocyte ratio” or “platelet to lymphocyte ratio” or “platelet-lymphocyte ratio” or “platelet lymphocyte” and “CRC” or “colon neoplasm” or “rectal neoplasm” or “colorectal neoplasm” or “colorectal tumor” or “colorectal cancer” or “colorectal carcinoma”. There was no language restriction in our study. References of relevant studies and review articles were searched for potential eligible studies.

### Inclusion and exclusion criteria

The inclusion criteria in this meta-analysis study were as follows: (1) studies investigated the relationship between PLR and colorectal cancer prognosis or clinical characteristics; (2) the PLR was obtained from a preoperative peripheral blood test; (3) adequate data were provided to measure odds ratio (OR) and hazard ratios (HRs) with 95% confidence intervals (CIs). The exclusion criteria were as follows: (1) unrelated studies, animal experiment, cell experiment, literature reviews, comments, letters, meta analysis, or case reports; (2) studies without sufficient data for analysis; and (3) duplicated publications. When studies with overlapping cases were met, the study with the larger number of patients was included.

### Data collection and quality assessment

Relevant datas were professionally extracted by two authors independently, and disagreements were resolved through discussion with a third author. Data collected from each study included first author, publication year, country and ethnicity of the study participants, number of patients, tumor characteristics (stage, location, size, differentiation, lymphovascular invasion, treatment, recurrence), cut off value for high or low PLR, and survival data (OS/DFS/CSS/RFS). If some publications provided survival data by Kaplan-Meier curves indirectly, Engauge Digitizer version 4.1 was applied to extract the data. The quality of included articles were assessed using the Newcastle-Ottawa Scale (NOS) by two authors independently (Supplementary Table S2 showed the Newcastle-Ottawa quality assessment scale). The total scores of NOS ranged from 0 to 9, with higher scores indicating better quality. A high-quality study was defined as the study with ≥6 points on NOS.

### Statistical analysis

According to the cut-off values, PLR was devided into high or low level groups in each study, and the hazard ratio with the 95% confidence interval (high *vs* low level of PLR) were used to evaluate the relationship between PLR and long-term prognosis (OS/DFS/CSS/RFS). Odds ratio and 95%CI were pooled to access the role of PLR on clinical features of colorectal cancer. Statistical heterogeneity was evaluated by Q and I^2^ tests, and if the *p*-value < 0.1 or I^2^ > 50%, which suggested the existence of substantial heterogeneity, thus we used a random-effect model to calculate the pooled estimate. Otherwise, the fixed-effect model would be applied instead. The subgroup analyses were applied to explore the heterogeneity sources. Publication bias was evaluated using the Egger's weighted linear regression and Begg's regression method. A trim and fill method was used when significant publication bias existed. All statistical tests were two-sided, and a *p* value < 0.05 was considered to be statistically significant. All analyses were conducted by Stata 14.0 (STATA Corporation, College Station, TX, USA).

## SUPPLEMENTARY MATERIALS TABLES



## Abbreviations

CRC: colorectal cancer
PLR: platelet-lymphocyte ratio
HR: hazard ratio
OR: odds ratio
95% CI: 95% confidence interval
OS: overall survival
DFS: disease-free survival
RFS: recurrence-free survival
CSS: Cancer-specific survival
NOS: the Newcastle-Ottawa Scale
Ph: P-value of heterogeneity
