# Supplementary material for: Increased platelet-lymphocyte ratio closely relates to inferior clinical features and worse long-term survival in both resected and metastatic colorectal cancer: an updated systematic review and meta-analysis of 24 studies
Source: Oncotarget. 2017 Mar 8;8(19):32356–69. doi: 10.18632/oncotarget.16020 (PMC5458290; doi:10.18632/oncotarget.16020)
Supplement: Supplementary file 1 [file oncotarget-08-32356-s001.doc]

Increased platelet-lymphocyte ratio closely relates to inferior clinical features and worse long-term survival in both resected and metastatic colorectal cancer: an updated systematic review and meta-analysis of 24 studies

**Supplementary Tables**

**Supplementary table S1** The extracted data on survival of included studies

| **First Author** | **Year** | **N^a^** | **Cut-off value** | **HR** | **95%CI** | |
| --- | --- | --- | --- | --- | --- | --- |
|  |  |  |  |  | **ll** | **ul** |
| **OS** |  |  |  |  |  |  |
| Azab | 2014 | 580 | >244 | 1.12 | 0.71 | 1.78 |
| Baranyai | 2014 | 336 | 300 | 3.5 | 2.2 | 5.6 |
| Baranyai | 2014 | 118 | 300 | 0.9 | 0.37 | 2.22 |
| Carruthers | 2012 | 115 | <160 | 1.5 | 0.8 | 2.7 |
| Chan | 2016 | 1623 | 258 | 1.592 | 1.343 | 1.886 |
| Choi | 2015 | 549 | 295 | 1.81 | 1.06 | 3.06 |
| He | 2013 | 243 | 300 | 1 | 0.79 | 1.27 |
| Kwon | 2012 | 200 | ≥300 | 1.953 | 1.161 | 3.284 |
| Li | 2016 | 140 | 144 | 1.697 | 1.064 | 2.708 |
| Li | 2016 | 5336 | 219 | 1.175 | 0.946 | 1.46 |
| Li | 2015 | 110 | 162 | 2.27 | 1.32 | 4.03 |
| Neal | 2015 | 302 | <150,150–300,>300 | 1.244 | 1.015 | 1.525 |
| Neofytou | 2014 | 140 | 150 | 2.17 | 1.09 | 4.32 |
| Passardi | 2016 | 289 | 169 | 1.27 | 0.89 | 1.8 |
| Son | 2013 | 624 | 300 | 2.006 | 0.53 | 7.589 |
| Song | 2015 | 177 | > 300 | 1.989 | 1.253 | 3.157 |
| Sun | 2014 | 255 | <150,150–300,>300 | 0.825 | 0.56 | 1.215 |
| Szkandera | 2014 | 372 | 225 | 1.49 | 0.92 | 2.4 |
| Toiyama | 2013 | 84 | 150 | 1.89 | 0.7 | 5.11 |
| Ying | 2014 | 205 | 176 | 1.15 | 0.77 | 1.73 |
| You | 2016 | 1314 | >220 vs ≤100 | 1.511 | 1.103 | 2.07 |
| Zou | 2016 | 216 | 246.36 | 2.029 | 1.077 | 3.821 |
| **DFS** |  |  |  |  |  |  |
| Azab | 2014 | 580 | >244 | 1.35 | 0.9 | 2.03 |
| Baranyai | 2014 | 336 | 300 | 3.4 | 2 | 5.8 |
| Carruthers | 2012 | 115 | <160 | 1.2 | 0.7 | 2.1 |
| Li | 2016 | 140 | 144 | 1.706 | 1.069 | 2.721 |
| Li | 2016 | 5336 | 219 | 1.073 | 0.92 | 1.251 |
| Mori | 2015 | 157 | 150 | 2.73 | 1.18 | 7.41 |
| Neofytou | 2014 | 140 | 150 | 1.68 | 1.04 | 2.71 |
| Ozawa | 2015 | 234 | 25.4 | 2.65 | 1.26 | 5.45 |
| Son | 2013 | 624 | 300 | 1.836 | 0.597 | 5.643 |
| Sun | 2014 | 255 | <150,150–300,>300 | 0.835 | 0.604 | 1.154 |
| Toiyama | 2013 | 84 | 150 | 1.66 | 0.67 | 4.09 |
| Zou | 2016 | 216 | 246.36 | 1.356 | 0.97 | 1.896 |
| **CSS** |  |  |  |  |  |  |
| Neal | 2015 | 302 | <150,150–300,>300 | 1.244 | 1.003 | 1.542 |
| Neofytou | 2015 | 140 | 150 | 1.006 | 1.002 | 1.009 |
| Ozawa | 2015 | 234 | 25.4 | 3.61 | 1.08 | 12.64 |
| Ying | 2014 | 205 | 176 | 1.15 | 0.75 | 1.78 |
| **RFS** |  |  |  |  |  |  |
| Choi | 2015 | 549 | 295 | 1.68 | 1.06 | 2.65 |
| Ying | 2014 | 205 | 176 | 1.21 | 0.82 | 1.77 |

^a^ Number of included patients

OS: overall survival; DFS: disease-free survival; CSS: Cancer-specific survival; RFS: recurrence-free survival; HR: hazard ratio; 95%CI: confidence interval; ll: lower limit; ul: upper limit.

**Supplementary table S2** The extracted data on clinical characteristics of included studies

| **Study** | **Year** | **Case** | | **Control** | |  | | | |
| --- | --- | --- | --- | --- | --- | --- | --- | --- | --- |
|  |  | **High level^b^** | **Low level^b^** | **High level^b^** | **Low level^b^** |  | | | |
| **Gender** |  | **Male** | | **Female** | |  | | | |
| Azab | 2014 | 83 | 190 | 112 | 195 |  | | | |
| Choi | 2015 | 26 | 270 | 25 | 228 |  | | | |
| He | 2013 | 16 | 139 | 6 | 82 |  | | | |
| Kwon | 2012 | 8 | 115 | 6 | 71 |  | | | |
| Li | 2016 | 43 | 44 | 41 | 24 |  | | | |
| Mori | 2015 | 34 | 54 | 24 | 28 |  | | | |
| Neofytou | 2014 | 27 | 116 | 26 | 65 |  | | | |
| Ozawa | 2015 | 19 | 43 | 13 | 9 |  | | | |
| Passardi | 2016 | 56 | 88 | 23 | 38 |  | | | |
| Toiyama | 2013 | 428 | 2739 | 350 | 1819 |  | | | |
| Ying | 2014 | 23 | 114 | 21 | 58 |  | | | |
| You | 2016 | 153 | 632 | 107 | 422 |  | | | |
| Zou | 2016 | 55 | 60 | 64 | 51 |  | | | |
| **Location** |  | **Colon** | | **Rectum** | |  | | | |
| Choi | 2015 | 47 | 421 | 4 | 75 |  | | | |
| He | 2013 | 14 | 161 | 8 | 60 |  | | | |
| Kwon | 2012 | 10 | 94 | 4 | 92 |  | | | |
| Li | 2016 | 43 | 139 | 10 | 42 |  |  |  |  |
| Ozawa | 2015 | 59 | 81 | 20 | 45 |  | | | |
| Passardi | 2016 | 410 | 1757 | 334 | 2690 |  | | | |
| Ying | 2014 | 26 | 87 | 18 | 85 |  | | | |
| You | 2016 | 112 | 328 | 96 | 601 |  | | | |
| Zou | 2016 | 110 | 102 | 44 | 33 |  | | | |
| **Differentiation** |  | **Poorly differentiated** | | **Well and moderately differentiated** | |  | | | |
| Choi | 2015 | 5 | 24 | 46 | 473 |  | | | |
| Kwon | 2012 | 0 | 16 | 14 | 170 |  | | | |
| Li | 2016 | 19 | 40 | 25 | 132 |  | | | |
| Mori | 2015 | 79 | 269 | 181 | 785 |  | | | |
| Ozawa | 2015 | 8 | 7 | 140 | 76 |  | | | |
| Toiyama | 2013 | 6 | 7 | 47 | 174 |  | | | |
| You | 2016 | 6 | 5 | 26 | 47 |  | | | |
| Zou | 2016 | 76 | 295 | 571 | 3619 |  | | | |
| **Stage** |  | **III/IV** | | **I/II** | |  | | | |
| Azab | 2014 | 85 | 141 | 95 | 201 |  | | | |
| Choi | 2015 | 13 | 172 | 38 | 324 |  | | | |
| Kwon | 2012 | 9 | 87 | 5 | 99 |  | | | |
| Mori | 2015 | 33 | 19 | 51 | 49 |  | | | |
| Ying | 2014 | 56 | 63 | 23 | 63 |  | | | |
| You | 2016 | 25 | 82 | 19 | 90 |  | | | |
| Zou | 2016 | 124 | 477 | 136 | 577 |  | | | |
| **T** |  | **3,4** | | **1,2** | |  | | | |
| Choi | 2015 | 41 | 332 | 10 | 161 |  | | | |
| Kwon | 2012 | 13 | 142 | 1 | 44 |  | | | |
| Li | 2016 | 49 | 41 | 35 | 27 |  | | | |
| Mori | 2015 | 68 | 114 | 3 | 20 |  | | | |
| Toiyama | 2013 | 636 | 3213 | 77 | 1092 |  | | | |
| Ying | 2014 | 42 | 144 | 2 | 28 |  | | | |
| Zou | 2016 | 27 | 41 | 5 | 11 |  | | | |
| **N** |  | **Positive(N1,2)** | | **Negative(N0)** | |  | | | |
| Choi | 2015 | 13 | 172 | 38 | 324 |  | | | |
| Kwon | 2012 | 9 | 86 | 5 | 100 |  | | | |
| Li | 2016 | 431 | 2145 | 346 | 2409 |  | | | |
| Toiyama | 2013 | 24 | 79 | 20 | 93 |  | | | |
| Ying | 2014 | 9 | 16 | 23 | 36 |  | | | |
| Zou | 2016 | 41 | 54 | 38 | 72 |  | | | |
| **LVI** |  | **Yes** | | **No** | |  | | | |
| Choi | 2015 | 15 | 140 | 35 | 345 |  | | | |
| Kwon | 2012 | 13 | 171 | 1 | 15 |  | | | |
| Li | 2016 | 111 | 81 | 141 | 123 |  | | | |
| Mori | 2015 | 52 | 144 | 54 | 218 |  | | | |
| Ozawa | 2015 | 224 | 1082 | 512 | 3160 |  | | | |
| You | 2016 | 39 | 146 | 221 | 908 |  | | | |
| **Recurrence** |  | **Present** | | **Absent** | |  | | | |
| Mori | 2015 | 22 | 6 | 62 | 62 |  | | | |
| Toiyama | 2013 | 8 | 8 | 24 | 44 |  | | | |
| **Chemotherapy** |  | **Yes** | | **No** | |  | | | |
| Azab | 2014 | 56 | 123 | 139 | 262 |  | | | |
| Choi | 2015 | 10 | 131 | 41 | 367 |  | | | |
| Li | 2016 | 44 | 64 | 35 | 62 |  | | | |
| Ying | 2014 | 636 | 3392 | 142 | 1166 |  | | | |

^a^Number of patients

^b^ Number of patients with low PLR level or high PLR level

LVI: lymphovascular invasion;

**Supplementary table S3** Newcastle - Ottawa quality assessment scale for cohort studies

NEWCASTLE - OTTAWA QUALITY ASSESSMENT SCALE

COHORT STUDIES

Note: A study can be awarded a maximum of one star for each numbered item within the Selection and Outcome categories. A maximum of two stars can be given for Comparability

**Selection (Max 4 Stars)**

1) Representativeness of the exposed cohort

a) truly representative of the average elderly, community-dwelling resident*

b) somewhat representative of the average, elderly, community-dwelling resident *

c) selected group of users eg nurses, volunteers

d) no description of the derivation of the cohort

2) Selection of the non exposed cohort

a) drawn from the same community as the exposed cohort *

b) drawn from a different source *

c) no description of the derivation of the non exposed cohort

3) Ascertainment of exposure

a) secure record (eg surgical records) *

b) structured interview *

c) written self report

d) no description

4) Demonstration that outcome of interest was not present at start of study

a) yes *

b) no

**Comparability** **(Max 2 Stars)**

1) Comparability of cohorts on the basis of the design or analysis

a) study controls for age, sex, marital status *

b) study controls for any additional factor * (This criteria could be modified to indicate specific control for a second important factor.)

**Outcome (Max 3 Stars)**

1) Assessment of outcome

a) independent blind assessment *

b) record linkage *

c) self report

d) no description

2) Was follow-up long enough for outcomes to occur

a) yes, if median duration of follow-up >= 6 month*

b) no, if median duration of follow-up < 6 months

3) Adequacy of follow up of cohorts

a) complete follow up - all subjects accounted for *

b) subjects lost to follow up unlikely to introduce bias - number lost <= 20%, or description of those lost suggesting no different from those followed *

c) follow up rate < 80% and no description of those lost

d) no statement

**Supplementary table S4** The detailed NOS scores of included studies

| First author | Publication year | NEWCASTLE - OTTAWA QUALITY ASSESSMENT SCALE | | | | | | | | |
| --- | --- | --- | --- | --- | --- | --- | --- | --- | --- | --- |
|  |  | Selection | | | | Comparabilitiy | Outcome | | | Total |
|  |  | Q1 | Q2 | Q3 | Q4 | Q1 | Q1 | Q2 | Q3 |  |
| Azab | 2014 | 1 | 1 | 1 | 1 | 2 | 1 | 1 | 0 | 8 |
| Baranyai | 2014 | 1 | 1 | 1 | 0 | 0 | 1 | 1 | 0 | 5 |
| Carruthers | 2012 | 1 | 1 | 1 | 1 | 0 | 1 | 1 | 0 | 6 |
| Chan | 2016 | 1 | 1 | 1 | 1 | 1 | 1 | 1 | 1 | 8 |
| Choi | 2015 | 1 | 1 | 1 | 1 | 2 | 1 | 1 | 0 | 8 |
| He | 2013 | 1 | 1 | 1 | 1 | 2 | 1 | 1 | 0 | 8 |
| Kwon | 2012 | 1 | 1 | 1 | 1 | 1 | 1 | 1 | 1 | 8 |
| Li | 2016 | 1 | 1 | 1 | 1 | 1 | 1 | 1 | 0 | 7 |
| Li | 2016 | 1 | 1 | 1 | 1 | 0 | 1 | 1 | 0 | 6 |
| Li | 2015 | 1 | 1 | 1 | 1 | 0 | 1 | 1 | 1 | 7 |
| Mori | 2015 | 1 | 1 | 1 | 1 | 0 | 1 | 1 | 0 | 6 |
| Neal | 2015 | 1 | 1 | 1 | 1 | 0 | 1 | 1 | 1 | 7 |
| Neofytou | 2014 | 1 | 1 | 1 | 1 | 2 | 1 | 1 | 1 | 9 |
| Neofytou | 2015 | 1 | 1 | 1 | 1 | 2 | 1 | 1 | 1 | 9 |
| Ozawa | 2015 | 1 | 1 | 1 | 1 | 1 | 1 | 1 | 0 | 7 |
| Passardi | 2016 | 1 | 1 | 1 | 0 | 2 | 1 | 1 | 0 | 7 |
| Son | 2013 | 1 | 1 | 1 | 1 | 0 | 1 | 1 | 1 | 7 |
| Song | 2015 | 0 | 1 | 1 | 1 | 0 | 1 | 1 | 0 | 5 |
| Sun | 2014 | 1 | 1 | 1 | 1 | 0 | 1 | 1 | 1 | 7 |
| Szkandera | 2014 | 1 | 1 | 1 | 0 | 1 | 1 | 1 | 1 | 7 |
| Toiyama | 2013 | 1 | 1 | 1 | 0 | 1 | 1 | 1 | 0 | 6 |
| Ying | 2014 | 1 | 1 | 1 | 1 | 1 | 1 | 1 | 0 | 7 |
| You | 2016 | 1 | 1 | 1 | 0 | 1 | 1 | 1 | 0 | 6 |
| Zou | 2016 | 1 | 1 | 1 | 1 | 1 | 1 | 1 | 0 | 7 |

Q:question.
